# Analysis of miRNA signatures in CSF identifies upregulation of miR-21 and miR-146a/b in patients with multiple sclerosis and active lesions

**DOI:** 10.1186/s12974-019-1590-5

**Published:** 2019-11-14

**Authors:** María Muñoz-San Martín, Gemma Reverter, Rene Robles-Cedeño, Maria Buxò, Francisco José Ortega, Imma Gómez, Jordi Tomàs-Roig, Naiara Celarain, Luisa María Villar, Hector Perkal, José Manuel Fernández-Real, Ester Quintana, Lluís Ramió-Torrentà

**Affiliations:** 1 Neuroimmunology and Multiple Sclerosis Unit, Neurology Department, Dr. Josep Trueta University Hospital, Girona Biomedical Research Institute (IDIBGI), Girona, Spain; 2 REEM. Red Española de Esclerosis Múltiple, Madrid, Spain; 3 Medical Sciences Department, Faculty of Medicine, University of Girona, Girona, Spain; 4 Girona Biomedical Research Institute (IDIBGI), Girona, Spain; 5 Department of Diabetes, Endocrinology and Nutrition, Girona Biomedical Research Institute (IDIBGI), Girona, Spain; 6 Immunology Department, Hospital Ramón y Cajal, IRYCIS, Madrid, Spain

**Keywords:** Active lesions, Epigenetics, Gadolinium positive (Gd+), Inflammation, miRNAs, Multiple sclerosis

## Abstract

**Background:**

MicroRNAs (miRNAs) have been reported as deregulated in active brain lesions derived from patients with multiple sclerosis (MS). In there, these post-transcriptional regulators may elicit very important effects but proper identification of miRNA candidates as potential biomarkers and/or therapeutic targets is scarcely available.

**Objective:**

The aim of the study was to detect the presence of a set of candidate miRNAs in cell-free cerebrospinal fluid (CSF) and to determine their association with gadolinium-enhancing (Gd+) lesions in order to assess their value as biomarkers of MS activity.

**Methods:**

Assessment of 28 miRNA candidates in cell-free CSF collected from 46 patients with MS (26 Gd+ and 20 Gd− patients) was performed by TaqMan assays and qPCR. Variations in their relative abundance were analyzed by the Mann-Whitney *U* test and further evaluated by receiver operating characteristic (ROC) analysis. Signaling pathways and biological functions of miRNAs were analyzed using bioinformatic tools (miRTarBase, Enrichr, REVIGO, and Cytoscape softwares).

**Results:**

Seven out of 28 miRNA candidates were detected in at least 75% of CSF samples. Consistent increase of miR-21 and miR-146a/b was found in Gd+ MS patients. This increase was in parallel to the number of Gd+ lesions and neurofilament light chain (NF-L) levels. Gene Ontology enrichment analysis revealed that the target genes of these miRNAs are involved in biological processes of key relevance such as apoptosis, cell migration and proliferation, and in cytokine-mediated signaling pathways.

**Conclusion:**

Levels of miR-21 and miR-146a/b in cell-free CSF may represent valuable biomarkers to identify patients with active MS lesions.

## Introduction

Multiple sclerosis (MS) is an immune-mediated disease of the central nervous system (CNS) characterized by inflammation, demyelination, and neurodegeneration. Inflammation of the white and gray matter tissues in the CNS are responsible for MS lesions, which are an evidence of the nerve cell damage produced in the brain or spinal cord [1, 2]. Disease activity in MS is strongly linked to the formation of new lesions that could be detected as a focal area of contrast enhancement on T1-weighted images obtained after gadolinium injection (Gd+) [3].

MicroRNAs (miRNAs) are small non-coding single-stranded RNA molecules with the ability to regulate gene expression at the post-transcriptional level by binding to target messenger RNAs, leading to their degradation or translational repression [4]. miRNAs may control many biological processes in health and disease including neurologic disorders such as MS [5]. Several studies in MS have analyzed the role or profile of miRNAs in different tissues including peripheral blood mononuclear cells (PBMCs) [6], CD4+ cells [7], and MS brain lesions [8]. They can be also released extracellularly into body fluids such as plasma or cerebrospinal fluid (CSF), where they remain stable [9–12].

CSF is in direct contact with the extracellular space of the brain and can mirror biochemical changes affecting the brain [13]. Recently, some studies have evaluated the presence of miRNAs in CSF and their usefulness as potential biomarkers of MS [14–16].

This study aims to test the presence of a set of deregulated miRNAs, previously found in active MS lesions from brain biopsies [8], in cell-free CSF of MS patients, and to study their association with the presence of gadolinium-enhancing (Gd+) lesions to assess their value as biomarkers of new MS lesion formation.

## Patients and methods

### Study design and patients

Patients included in this study were recruited at the Girona Neuroimmunology and Multiple Sclerosis Unit of Dr. Josep Trueta University Hospital (Girona, Spain) and in the Immunology and Neurology Departments of Ramón y Cajal University Hospital (Madrid, Spain). A sample of 46 relapse-onset MS patients over 18 years old was included. Twenty-six MS patients showed Gd+ lesions in their magnetic resonance imaging (MRI) (Gd+), whereas 20 patients were not characterized by this clinical output (Gd−). CSF and plasma samples were collected at the diagnosis of the disease. MRI acquisition was made within 30 days from the sample collection. All patients were naïve of disease-modifying drugs. Demographic data including sex ratio and age, as well as clinical and radiological outputs, are depicted in Table 1. The Ethics Committee and the Committee for Clinical Investigation from Dr. Josep Trueta University Hospital approved the protocol employed in this study. All participants signed a written informed consent.

**Table 1:** Demographic data of the study cohort

	Gd− (*n* = 20)	Gd+ (*n* = 26)	*p* value
Age (mean ± SD)	31.15 ± 10.37	36.15 ± 8.65	0.081
Sex (male/female)			0.818
Male	6 (30.00%)	7 (26.92%)	
Female	14 (70.00%)	19 (73.08%)	
EDSS at sampling (median, Q1–Q2)	2 (1–2.5)	2 (1.5–2.13)	0.819
OCGB (N/P)			0.432
Negative	1 (5.26%)	0 (0.00%)	
Positive	18 (94.74%)	25 (100.00%)	
LS_OCMB (N/P)			0.393
Negative	14 (73.68%)	16 (61.54%)	
Positive	5 (26.32%)	10 (38.46%)	
T2 lesions			0.004
< 9 lesions	12 (66.67%)	4 (16.67%)	
10–20 lesions	5 (27.78%)	10 (41.67%)	
21–50 lesions	1 (5.56%)	8 (33.33%)	
51–100 lesions	0 (0.00%)	2 (8.33%)	

*Gd−* patients without gadolinium-enhanced lesions, *Gd+* patients with gadolinium-enhanced lesions; *SD* standard deviation, *OCBG* oligoclonal IgG bands; *N/P* negative/positive, *LS_OCMB* lipid-specific oligoclonal IgM bands

### Samples

Following consensus conditions for CSF and plasma collection and biobanking, CSF was centrifuged immediately after a lumbar puncture at 400×*g* for 15 min to obtain cell-free CSF. Plasma was collected in EDTA tubes and centrifuged at 2000×*g* for 10 min. Cell-free CSF and plasma aliquots were stored at − 80 °C until RNA extraction [17].

### Analysis of circulating miRNAs

#### Circulating RNA extraction and purification

Total RNA was extracted from CSF or plasma using mirVana PARIS Isolation Kit (Applied Biosystems) according to the manufacturer’s instructions. In brief, 300 μl of sample was mixed with an equal volume of 2× Denaturing solution. Spike-in cel-miR-39 and cel-miR-54 exogenous miRNAs were added to check the quality of the extraction. Then, the same volume of acid-phenol:chloroform was added. After centrifugation (17.000×*g*, 10 min, 19 °C), the upper aqueous phase was recovered and mixed with 100% ethanol. After being placed into a filter cartridge, three washing steps were performed. Total RNA was eluted with 40 μl of nuclease-free water.

#### Circulating miRNA RT and preamplification

We used a fixed volume of 3 μl of total purified for the reverse transcription (RT). A set of 754 miRNAs were processed in CSF samples, using TaqMan miRNA Reverse Transcription Kit and Multiplex RT Assays (Human pool sets A and B) (Applied Biosystems) as previously described [18]. For RNA isolated from plasma samples, we used custom RT primer pool directed to the miRNAs identified as altered in CSF following the appropriate protocol. Each protocol has demonstrated sensitivity, specificity as well as repeatable and accurate results [19]. RT products for both CSF and plasma were then preamplified by means of TaqMan PreAmp Master Mix and/or Megaplex PreAmp Primers (Human pool sets A and B) (Applied Biosystems), or by custom preamplification primer pool, respectively. Preamplification step is mandatory before real-time PCR (RT-PCR) reaction when analytical sensitivity is the utmost importance and the sample is limiting [18].

#### Analysis of individual miRNAs with TaqMan hydrolysis probes

To study the presence of circulating miRNA candidates, the preamplification product was diluted (1:50 for CSF and 1:200 for plasma) and quantified with Individual TaqMan hydrolysis probes (Applied Biosystems). Individual gene expression was assessed by RT-PCR using the Light-Cycler® 480 System (Roche Molecular Biochemicals) and triplicates for each sample were performed. Expression values in CSF were normalized using miR-17 as previously described [16]. Whereas plasma levels were normalized using the mean value of miR-17, miR-191, miR-103, and miR-186.

### Targets and pathway analyses

Experimentally validated targets for differentially expressed miRNAs were retrieved from miRTarBase [20]. Those genes which validation was made by western blot, reporter assay and/or qPCR were introduced in Enrichr to explore the gene ontology (GO) biological processes [21]. Associated GO terms were clustered according to their relatedness using REVIGO [22] after removing redundant terms. A microRNA-target interaction network was performed using Cytoscape software [23].

### NF-L quantification

Levels of neurofilament light chain (NF-L) in CSF of 25 patients were measured by ELISA (UmanDiagnostics, Umea, Sweden) at the Ramón y Cajal University Hospital (Madrid, Spain) according to the manufacturer’s instructions.

### Microarray data analysis

We downloaded publically available gene expression datasets, containing normalized data, from a study which contains gene and miRNA expression levels from human immune cell subsets [24]. We wanted to study the possible interaction between our deregulated miRNAs and their shared targets in silico. The datasets were included in the SuperSeries GSE28492.

### Statistical analysis

Demographic, clinical, radiological, and normalized mRNA levels data were reported as follows: categorical variables were shown as frequencies and percentages while continuous variables were represented by mean ± standard deviation (SD) or median (quartiles). Normal distribution of a data set was assessed with normal Q-Q plots and the Shapiro-Wilk test. Statistical differences were determined using Student’s *t* test and Mann-Whitney *U* test in the case of quantitative variables while chi-square test or Fisher’s exact test for categorical ones. Correlation analysis of normalized miRNA levels and clinical/radiological/biochemical data were estimated by Spearman’s Rho (*r*_*s*_). Receiver operating characteristic (ROC) analysis was conducted to determine the ability of miRNA levels to discriminate between patients with Gd+ and Gd− lesions. Area under the curve (AUC), 95% confidence interval (95% CI), and the optimal cut-off values using Youden’s index were determined. The sensitivity, specificity, and positive and negative likelihood ratios (LR+ and LR−, respectively) were also calculated. In addition, a ROC curve for combined miRNAs was calculated via logistic analysis. All analyses were two-tailed and the significance level was set to 0.05. Statistical analyses were performed in Statistical Package for the Social Sciences (SPSS) version 25.0 (IBM SPSS Statistics for Windows, NY, USA) and R 3.4.3 using pROC package [25]. Figures were built with GraphPad PRISM v.5.

## Results

### Detection of miRNAs in CSF

Forty-six MS patients (71.7% women) with a mean age and standard deviation of 33.98 ± 9.66 were studied. Gd+ lesions were present in 56.5% of the subjects. No differences were observed for either sex distribution or age between Gd− and Gd+ patients (Table 1). Seven of 28 miRNAs reported as deregulated in active MS lesions (9) were detected in at least 75% of CSF samples, assessed in triplicate by Ct values lower than 37 (miR-155, miR-223, miR-21, miR-320, miR-328, miR-146a, and miR-146b). A group of five miRNAs was present in less than 75% of samples (miR-34a, miR-130a, miR-214, miR-27a, and miR-656) while the others show very low expression measures or were undetected (Additional file 1: Table S1).

### Differential miRNA values in CSF of MS patients with Gd+ lesions

**Fig. 1:**
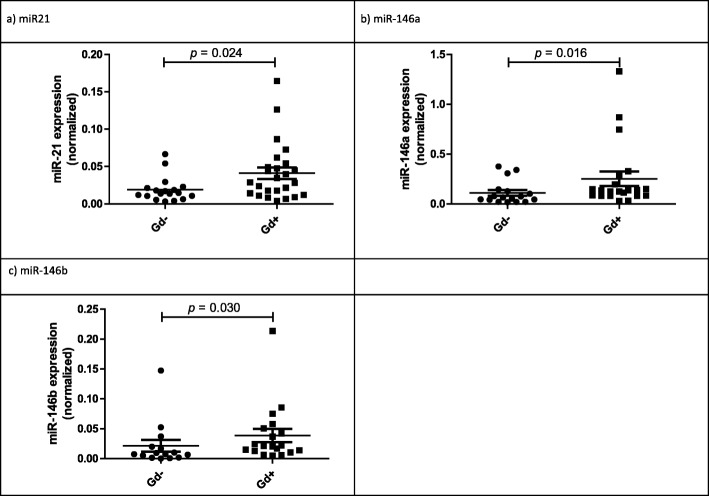
Differentially expressed miRNAs. Dot plots for normalized value of **a** miR-21, **b** miR-146a, and **c** miR-146b in patients according to the presence of Gd+ lesions in the brain. The line indicates the median and Mann-Whitney *U* test was used to determine statistical differences between groups

Differential expression of the seven miRNAs detected in at least 75% of the CSF samples was tested to compare Gd− and Gd+ patients. Increased expression of miR-21, miR-146a, and miR-146b was found in Gd+ patients (Table 2 and Fig. 1). ROC curves were performed to determine the potential discriminatory capacity provided by each single miRNA present in cell-free CSF for detecting brain acute inflammatory activity. miR-21, miR-146a, and miR-146b presented AUC values of 0.703, 0.728, and 0.712, respectively, which support a valuable discriminatory indicator to differentiate Gd− from Gd+ patients. Logistic regression analysis was performed to evaluate the discriminatory ability of selected miRNAs combination. Variables were dichotomized according to cut-off values deduced from ROC analysis (AUC = 0.867) (Table 3, Additional file 1: Figure S1).

**Table 2:** Differential miRNA expression between groups in CSF

	Gd*−* (*n* = 20)		Gd + (*n* = 26)		
	Median	Q1-Q3	Median	Q1–Q3	*p* value
miR-21	0.014	0.010–0.022	0.029	0.013–0.052	0.024
miR-146a	0.065	0.031–0.136	0.135	0.082–0.239	0.016
miR-146b	0.008	0.002–0.020	0.021	0.012–0.052	0.030
miR-155	0.007	0.002–0.016	0.011	0.006–0.019	0.163
miR-223	0.066	0.043–0.328	0.124	0.088–0.226	0.232
miR-320	0.038	0.010–0.048	0.042	0.026–0.087	0.271
miR-328	0.007	0.003–0.012	0.011	0.005–0.026	0.143

*Gd−* patients without gadolinium-enhanced lesions, *Gd+* patients with gadolinium-enhanced lesions; *Q1–Q3* first quartile–third quartile

**Table 3:** Capacity of selected miRNAs to detect inflammatory activity in CNS

	AUC	95% CI	Sensitivity (%)	Specificity (%)	LR+	LR−
miR-21	0.703	0.543–0.863	60.00	77.78	2.70	0.51
miR-146a	0.728	0.553–0.904	90.48	64.71	2.56	0.15
miR-146b	0.712	0.531–0.894	81.82	66.67	2.45	0.27
miR-21 + miR-146a + miR-146b	0.867	0.736–0.997	94.12	69.23	3.06	0.08

*AUC* area under curve, *CI* confidence interval, *LR+* positive likelihood ratio, *LR*− negative likelihood ratio

### Correlation between deregulated miRNAs and CSF NF-L levels

Differential expression of NF-L levels was tested to compare Gd− and Gd+ subjects (data not shown). Increased expression of NF-L was found in CSF of Gd+ patients (median Gd− = 462.5 ng/L vs median Gd+ = 1116.0 ng/L; *p* = 0.010).

**Fig. 2:**
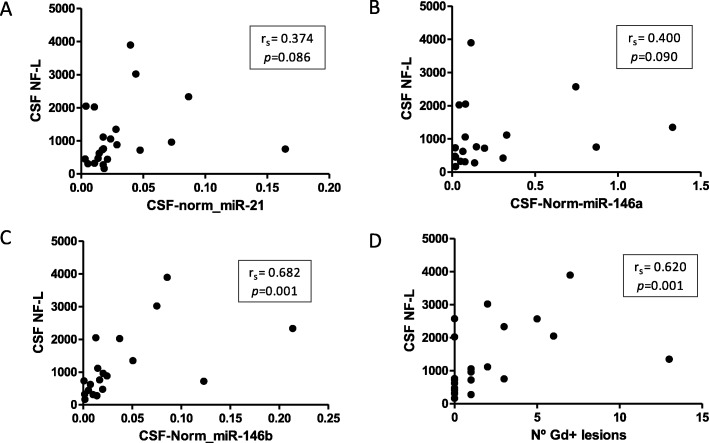
Scatter plot of correlation between NF-L in CSF. Scatter plots showing the relationship between NF-L levels in CSF and expression levels of miR-21 (**a**), miR-146a (**b**), miR-146b (**c**), and number of Gd+ lesions (**d**). Significant positive correlations were observed in all cases, illustrating a link between axonal damage and candidate miRNAs expression. *r*_s_: Rho Spearman; *p*: *p* value

We also studied the correlation between CSF deregulated miRNA levels and NF-L levels (Fig. 2). The expression of miR-146b was strongly positively correlated with NF-L (*r*_s_ = 0.682 and *p* = 0.001) while miR-21 and miR-146a showed a moderate relationship (*r*_s_ = 0.374 and *p* = 0.086, *r*_s_ = 0.400 and *p* = 0.090, respectively). NF-L levels also presented strong correlation with number of Gd+ lesions (*r*_s_ = 0.620 and *p* = 0.001) (Fig. 2).

**Fig. 3:**
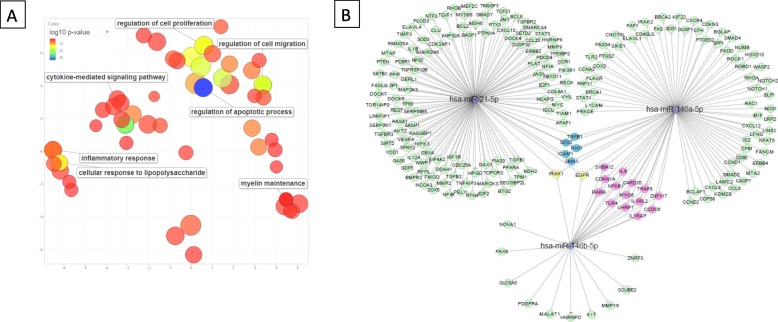
Pathway and target analysis of miR-21, miR-146a, and miR-146b. **a** Visualization of the significantly associated GO biological processes using REVIGO. The semantic similarity-scatterplot shows the cluster representatives after the redundancy reduction. The bubble size indicates the frequency of the GO term and the color indicates the log_10_ (adjusted *p* value). Color legend is shown in the left corner of the panel **b**: microRNA-target interaction network generated by Cytoscape software. Targets with strong experimental evidence for miR-21, miR-146a, and miR-146b were retrieved from miRTarBase [20]. Blue-colored targets correspond to those targets shared by miR-21 and miR-146a; pink-colored targets corresponds to those targets shared by miR-146a and miR-146b; yellow-colored targets corresponds to those targets shared by the three deregulated miRNAs

### Association of CSF miRNA expression with radiological and clinical variables

Levels of miR-21, miR-146a, and miR-146b in CSF positively correlated with the number of Gd+ lesions. In addition, the expression of miR-21 and miR-155 mirrored the number of lesions in T2 weighted images. Finally, miR-21 was also associated with the basal expanded disability status scale (EDSS) score (Table 4).

**Table 4:** Correlations between radiological and clinical data with miRNAs expression

	No. of Gd+ lesions		No. of T2 lesions		Basal EDSS	
	*r*_p_	*p* value	*r*_p_	*p* value	*r*_p_	*p* value
miR-21	0.379	0.015	0.377	0.017	0.440	0.003
miR-146a	0.463	0.004	0.125	0.474	0.030	0.856
miR-146b	0.349	0.040	0.004	0.984	− 0.299	0.072
miR-155	0.270	0.077	0.308	0.047	− 0.010	0.949
miR-223	0.159	0.309	0.113	0.481	0.249	0.099
miR-320	0.187	0.243	0.035	0.831	0.035	0.825
miR-238	0.298	0.073	0.213	0.211	− 0.089	0.592

*r*_s_ Spearman’s Rho coefficient

### Target and pathway analysis of miR-21, miR-146a, and miR-146b

Experimentally validated human molecules with strong evidence for being targets for miR-21, miR-146a, and miR-146b were retrieved from miRTarBase (Additional file 1: Table S2) and then, uploaded to Enrichr to explore the GO biological processes. Clustered GO terms revealed that miRNAs target genes were involved in distinct biological processes such as apoptosis, cell migration, and proliferation and cytokine-mediated signaling pathways (Fig. 3a). The miRNA-target interaction network showed interleukin receptor-associated kinase 1 (*IRAK1*) and epidermal growth factor receptor *(EGFR)* as shared targets for the three deregulated miRNAs (Fig. 3b).

### Correlation analysis of miRNAs and mRNA expression in in silico datasets

In order to evaluate the possible interaction between miR-21, miR-146a and miR-146b, and their shared targets *IRAK1* and *EGFR*, we used the normalized publicly available datasets GSE28487 (miRNA expression) and GSE28490 (mRNA expression) belonging to the SuperSeries GSE28492 from GEO [24]. We made correlation analysis between normalized miRNA levels and *IRAK1* and *EGFR* mRNA levels in the whole set of studied immune cells. We found that *IRAK1* was strongly negatively correlated to miR-146a and miR-146b expression (*r*_s_ = − 0.670 and *r*_s_ = − 0.635, respectively), while the relation with miR-21 did not reach statistical significance. However, the expression of *EGFR* was not correlated to the expression of these miRNAs (Additional file 1: Figure S2).

### Plasma expression of miR-21, miR-146a, and miR-146b

Circulating miRNAs from plasma samples of 30 patients included in the CSF study (13 Gd− and 17 Gd+ patients) were extracted and quantified by RT-PCR. Differential expression analysis of miRNAs in plasma based on Gd− or Gd+ status revealed no significant differences for the three deregulated miRNAs in CSF (Additional file 1: Table S3). Furthermore, no significant correlation between CSF and plasma miRNA levels was found, with the exception of miR-146b (*r*_s_ = − 0.398 and *p* = 0.049) (Additional file 1: Figure S3). Moreover, no association was observed between plasma miRNA levels and number of Gd+ lesions. Only plasma level of miR-146a showed a positive correlation with EDSS (*r*_s_ = 0.383 and *p* = 0.037)

## Discussion

Circulating miRNAs are being widely studied as potential biomarkers of diagnosis and prognosis in different diseases due to their stability and the ease to be measured in tissues and biological fluids [26]. Most of these studies are related to cancer research and have demonstrated the ability of circulating miRNAs as a new and reliable diagnosis and prognosis biomarker to detect and identify different cancer [27–29]. Recent studies elucidate the role of miRNAs in neurodegenerative diseases, such as MS, and their capacity to predict disease subtype with a high degree of accuracy [30, 31], as well as, response to a specific treatment [32]. Several studies have analyzed miRNA expression in cell-free CSF [14–16], a biological fluid that could mirror events occurring in the CNS. However, none of them have checked their relationship with the presence of active MS lesions. We hypothesized that the differential expression of miRNAs in CSF collected from MS patients could be a valuable indicator of CNS inflammation.

Junker et al. [8] reported a set of 28 miRNAs deregulated in brain tissue with active MS lesions. Our results confirmed the presence in the CSF of seven of the miRNAs previously reported as deregulated in active brain lesions (miR-21, miR-146a, miR-146b, miR-155, miR-223, miR-320, and miR-328). Moreover, miR-21, miR-146a, and miR-146b were overexpressed in the CSF of Gd+ MS patients and also associated with radiological variables and clinical disability. This overexpression also agrees with the findings reported in active MS lesions, where these three miRNAs were upregulated when compared to normal white matter [8]. We also have observed a correlation between miR-21, miR-146a and miR-146b, and CSF NF-L levels, one of the most validate biomarkers for axonal damage [33], what supports the relation between these miRNAs and injury in the CNS.

Fenoglio et al. [34] found miR-21 and miR-146a/b over-represented in PBMCs of relapsing-remitting MS patients compared to controls. They suggested that this upregulation was specific to the acute phase of MS and contribute to the differentiation and regulation of CD4+ T cells, which are involved in the CNS inflammatory processes that take place in MS [35]. First, activated T lymphocytes migrate from the periphery into the CNS through the blood-brain barrier. Then, CD4+ T cells contribute to maintain the inflammation within the CNS and cytotoxic CD8+ T cells can induce direct axonal damage [36]. However, regulatory T cells (T reg), which maintain immune self-tolerance by suppressing effector T cells, exert that control, in part, by the release of miRNA-containing exosomes as non-cell-autonomous gene silencing mechanism [37]. In addition, extracellular vesicles released by Treg cells are enriched in miR-21 and miR-146a [38].

These miRNAs have several roles in regulating T cell biology. miR-21 may modulate T cell activation and apoptosis, Treg function and development, or Th17 differentiation [39]. On the other hand, miR-146a is thought to constitute an important negative regulator of the innate immune response [40] and controls IL17 production [41]. Remarkably, the expression of miR-146b is increased upon the presence of proinflammatory cytokines as IFN-γ [42]. Thus, we could speculate that an overexpression of these miRNAs might be reactive to the proinflammatory milieu in MS. In the presence of CNS inflammatory processes miR-21, miR-146a and miR-146b could be highly expressed in order to counteract the harmful activity of other cells.

In silico pathway analysis underpins the relevance of these miRNAs in the acute phase of inflammation in the CNS of RRMS patients. Specifically, their target genes are involved in apoptosis, cell migration and proliferation, immune response, and cytokine-mediated signaling. As previously mentioned, migration of T lymphocytes into the CNS is a prerequisite to tissue damage in MS, and all these biological processes are involved in the cascade of events that trigger brain lesions. The miRNA-target interaction analysis revealed that the three overexpressed miRNAs have common targeted genes *IRAK1* and *EGFR* [43–47]*.* However, the in silico analysis of public datasets of miRNA and mRNA expression arrays showed that only *IRAK1* expression seemed to be negatively correlated with miR-146a and miR-146b levels mainly. IRAK1 is a serine/threonine kinase associated with interleukin-1 receptor (IL-1R) and Toll-like receptor (TLR) signaling pathways that play a role in the innate immune response and exert an important influence in T helper differentiation and proliferation [48]. IRAK1-deficient mice are resistant to experimentally induced autoimmune encephalomyelitis [49], suggesting a critical role of this protein in autoimmune and inflammatory diseases. The use of IRAK1 inhibitors was tested to treat inflammatory diseases [50]. Thus, even though the levels of circulating miRNAs do not necessarily reflect deregulation in cells and that the use of circulating miRNAs as biomarkers should not be confounded with their potential regulatory functions inside the cells, these in silico observations suggest that it would be interesting to study the modulation of *IRAK1* expression due to the overexpression of miR-21, miR-146a, and miR-146b in the CSF in order to prove its role in controlling inflammation and re-establish the self-tolerance after an acute inflammatory event.

Finally, due to the invasiveness of the lumbar puncture, the detection of biomarkers in serum or plasma is the most appropriate option for monitoring disease progression. Although we were able to detect miR-21, miR-146a, and miR-146b in plasma samples, we found no significant differences between Gd+ and Gd− patients. Other groups also failed to correlate their CSF and plasma findings [15, 51]. The poor correlation between CSF and plasma findings might suggest that CSF miRNA profile could provide different information not available in plasma.

## Conclusions

In conclusion, overexpression of miR-21, miR-146a, and miR-146b in cell-free CSF were able to discriminate MS patients with Gd+ lesions in the MRI. All data point to the hypothesis that an overexpression of these miRNAs in CSF is induced to counteract the pro-inflammatory milieu in MS, and they might be released into the CSF in an attempt to reduce the harmful damage into the brain. However, additional functional studies and analyses of larger cohorts are needed to validate these results and to elucidate the real role of these miRNAs in the context of MS.

## Supplementary information

**Additional file 1: Table S1.** List of 28 analyzed miRNAs and percentage of detection for each miRNAs in CSF. **Table S2.** Validated targets for miR-21-5p, miR-146a-5p and miR-146b-5p from miRTarBase. **Table S3.** Differential miRNA expression between groups in plasma. **Figure S1.** TITLE: Receiver Operating Characteristics (ROC) analysis of individual (a-c) and combined (d) CSF miR-21, miR-146a and miR-146b to discriminate inflammatory activity. **Figure S2.** Correlation analysis of deregulated miRNAs and candidate targets mRNA expression in publicly available dataset. miRNA and mRNA expression profile of GSE28487 and GSE28490, respectively from Gene Expression Omnibus (GEO) DataSets (https://www.ncbi. nlm.nih.gov/gds/) was obtained. Scatter plots of correlations between upregulated miRNAs in CSF (miR-21, miR-146a and miR-146b) and their shared target genes (*IRAK-1* and *EGFR*) were presented. r_s_: Rho Spearman; *p*: *p* value. **Figure S3.** Correlation of normalized Ct between CSF and plasma for (a) miR-21, (b) miR-146a and (c) miR-146b. Scatter plots showing the relationship between CSF and plasma levels for the deregulated miRNAs. r_s_: Rho Spearman; *p*: p value.

## Data Availability

Please contact the corresponding author for datasets requests.

## Abbreviations

95% CI: 95% confidence interval
AUC: Area under the curve
CNS: Central nervous system
CSF: Cerebrospinal fluid
EDSS: Expanded disability status scale
*EGFR*: Epidermal growth factor receptor
Gd−: Non-gadolinium-enhancing
Gd+: Gadolinium-enhancing
GO: Gene ontology
IL-1R: Interleukin-1 receptor
*IRAK1*: Interleukin receptor-associated kinase 1
LR: Likelihood ratio
miRNAs: MicroRNAs
MRI: Magnetic resonance imaging
MS: Multiple sclerosis
PBMCs: Peripheral blood mononuclear cells
ROC: Receiver operating characteristic
RT: Reverse transcription
RT-PCR: Real-time PCR
SD: Standard deviation
SPSS: Statistical Package for the Social Sciences
TLR: Toll-like receptor
