# Supplementary material for: Analysis of miRNA signatures in CSF identifies upregulation of miR-21 and miR-146a/b in patients with multiple sclerosis and active lesions
Source: J Neuroinflammation. 2019 Nov 14;16:220. doi: 10.1186/s12974-019-1590-5 (PMC6857276; doi:10.1186/s12974-019-1590-5)
Supplement: Supplementary file 1 — Additional file 1: Table S1. List of 28 analyzed miRNAs and percentage of detection for each miRNAs in CSF. Table S2. Validated targets for miR-21-5p, miR-146a-5p and miR-146b-5p from miRTarBase. Table S3. Differential miRNA expression between groups in plasma. Figure S1. TITLE: Receiver Operating Characteristics (ROC) analysis of individual (a-c) and combined (d) CSF miR-21, miR-146a and miR-146b to discriminate inflammatory activity. Figure S2. Correlation analysis of deregulated miRNAs and candidate targets mRNA expression in publicly available dataset. miRNA and mRNA expression profile of GSE28487 and GSE28490, respectively from Gene Expression Omnibus (GEO) DataSets (https://www.ncbi. nlm.nih.gov/gds/) was obtained. Scatter plots of correlations between upregulated miRNAs in CSF (miR-21, miR-146a and miR-146b) and their shared target genes (IRAK-1 and EGFR) were presented. rs: Rho Spearman; p: p value. Figure S3. Correlation of normalized Ct between CSF and plasma for (a) miR-21, (b) miR-146a and (c) miR-146b. Scatter plots showing the relationship between CSF and plasma levels for the deregulated miRNAs. rs: Rho Spearman; p: p value. [file 12974_2019_1590_MOESM1_ESM.docx]

Table S1. List of 28 analysed miRNAs and percentage of detection for each miRNAs in CSF

|  | Gd- patients  (*n* = 20) | |  | Gd+ patients  (*n* = 26) | | Total % |
| --- | --- | --- | --- | --- | --- | --- |
|  | *n* | % |  | *n* | % |  |
| miR-155 | 20 | 100.00 |  | 26 | 100.00 | 100.00 |
| miR-223 | 19 | 95.00 |  | 26 | 100.00 | 97.83 |
| miR-21 | 18 | 90.00 |  | 25 | 96.15 | 93.48 |
| miR-320 | 19 | 95.00 |  | 24 | 92.31 | 93.48 |
| miR-328 | 16 | 80.00 |  | 23 | 88.46 | 84.78 |
| miR-146a | 17 | 85.00 |  | 21 | 80.77 | 82.61 |
| miR-146b | 15 | 75.00 |  | 22 | 84.62 | 80.44 |
| miR-34a | 13 | 65.00 |  | 14 | 53.85 | 58.70 |
| miR-130a | 10 | 50.00 |  | 17 | 65.39 | 58.70 |
| miR-214 | 10 | 50.00 |  | 14 | 53.85 | 52.17 |
| miR-27a | 10 | 50.00 |  | 12 | 46.15 | 47.83 |
| miR-656 | 7 | 35.00 |  | 13 | 50.00 | 43.48 |
| miR-487b | 8 | 40.00 |  | 4 | 15.39 | 26.09 |
| miR-200c | 6 | 30.00 |  | 6 | 23.08 | 26.09 |
| miR-184 | 4 | 20.00 |  | 5 | 19.23 | 19.57 |
| miR-23a | 3 | 15.00 |  | 4 | 15.39 | 15.22 |
| miR-139 | 2 | 10.00 |  | 4 | 15.39 | 13.04 |
| miR-340 | 3 | 15.00 |  | 2 | 7.69 | 10.87 |
| miR-142-5p | 2 | 10.00 |  | 2 | 7.69 | 8.70 |
| miR-23b | 2 | 10.00 |  | 1 | 3.85 | 6.52 |
| miR-650 | 1 | 5.00 |  | 1 | 3.85 | 4.35 |
| miR-193a | 1 | 5.00 |  | 0 | 0.00 | 2.17 |
| miR-181c | 0 | 0.00 |  | 1 | 3.85 | 2.17 |
| miR-326 | 0 | 0.00 |  | 0 | 0.00 | 0.00 |
| miR-142-3p | 0 | 0.00 |  | 0 | 0.00 | 0.00 |
| miR-199a | 0 | 0.00 |  | 0 | 0.00 | 0.00 |
| miR-22 | 0 | 0.00 |  | 0 | 0.00 | 0.00 |
| miR-15a | 0 | 0.00 |  | 0 | 0.00 | 0.00 |

Table S2. Validated targets for miR-21-5p, miR-146a-5p and miR-146b-5p from miRTarBase.

| miR-21-5p | miR-21-5p | miR-146a-5p | miR-146a-5p | miR-146b-5p |
| --- | --- | --- | --- | --- |
| AKT2 | NTF3 | BCLAF1 | RARB | CARD10 |
| ANKRD46 | PCBP1 | BGLAP | RHO | CCDC6 |
| ANP32A | PDCD4 | BRCA1 | RHOA | CDKN1A |
| APAF1 | PELI1 | BRCA2 | RNF11 | EGFR |
| BASP1 | PIAS3 | CARD10 | ROBO1 | ERBB4 |
| BCL2 | PIK3R1 | CASP7 | ROCK1 | HNRNPD |
| BCL6 | PLAT | CCDC6 | S100A12 | IL1RAP |
| BMPR2 | PLOD3 | CCL5 | SIKE1 | IL1RL2 |
| BTG2 | PPARA | CCNA2 | SLPI | IL6 |
| CBX4 | PPIF | CCND1 | SMAD2 | IRAK1 |
| CCL20 | PTEN | CCND2 | SMAD4 | KIT |
| CCR1 | PTPN14 | CD40LG | SMN1 | MALAT1 |
| CDC25A | PTX3 | CD80 | SOS1 | MMP16 |
| CDK2AP1 | RASA1 | CDKN1A | SOX2 | MYO6 |
| CLU | RASGRP1 | CDKN3 | SPP1 | NFKB1 |
| COL4A1 | RECK | CFH | STAT1 | NOVA1 |
| CXCL10 | REST | CNOT6L | TGFB1 | PAX8 |
| DAXX | RFFL | COPS8 | TLR2 | PDGFRA |
| DDAH1 | RHO | COX2 | TLR4 | RARB |
| DERL1 | RHOB | CPM | TRAF6 | S100A12 |
| DOCK4 | RMND5A | CXCL12 | UHRF1 | SCUBE2 |
| DOCK5 | RPS7 | CXCL8 | WASF2 | SLC5A5 |
| DOCK7 | RTN4 | CXCR4 | ZNF117 | TLR4 |
| DUSP10 | SASH1 | DUSP1 |  | TRAF6 |
| E2F1 | SATB1 | EGFR |  | UHRF1 |
| E2F2 | SECISBP2L | ELAVL1 |  | ZNF117 |
| EGFR | SERPINB5 | ERBB4 |  | ZNRF3 |
| EIF4A2 | SERPINI1 | FADD |  |  |
| ELAVL4 | SETD2 | FAF1 |  |  |
| ERBB2 | SIRT2 | FANCM |  |  |
| FASLG | SMAD7 | FAS |  |  |
| FBXO11 | SMARCA4 | HOXD10 |  |  |
| FMOD | SMN1 | ICAM1 |  |  |
| GAS5 | SOD3 | IL1RAP |  |  |
| GDF5 | SOX2 | IL1RL2 |  |  |
| HIPK3 | SOX5 | IL6 |  |  |
| HNRNPK | SP1 | IRAK1 |  |  |
| HPGD | SPRY2 | IRAK2 |  |  |
| ICAM1 | STAT3 | IS2 |  |  |
| IGF1R | TCF21 | KDM2B |  |  |
| IL12A | TGFB1 | KIF22 |  |  |
| IL1B | TGFB2 | L1CAM |  |  |
| IRAK1 | TGFBI | LAMC2 |  |  |
| ISCU | TGFBR2 | LFNG |  |  |
| JAG1 | TGFBR3 | LIN52 |  |  |
| JMY | TGIF1 | LRP2 |  |  |
| LRRFIP1 | TIAM1 | MIF |  |  |
| MAP2K3 | TIMP3 | MTA2 |  |  |
| MARCKS | TM9SF3 | MYO6 |  |  |
| MEF2C | TNFAIP3 | NFAT5 |  |  |
| MMP2 | TNFRSF10B | NFKB1 |  |  |
| MMP9 | TOPORS | NOS1 |  |  |
| MSH2 | TOR1AIP2 | NOTCH1 |  |  |
| MSH6 | TP53BP2 | NOTCH2 |  |  |
| MTAP | TP63 | NUMB |  |  |
| MYC | TPM1 | PA2G4 |  |  |
| MYD88 | VEGFA | PLAUR |  |  |
| NCAPG | VHL | PRKCE |  |  |
| NCOA3 | WWP1 | PTGES2 |  |  |
| NFIA | YOD1 | PTGS2 |  |  |
| NFIB |  | RAC1 |  |  |

Table S3. Differential miRNA expression between groups in plasma

|  | Gd- (*n* = 13) | |  | Gd+ (*n* = 17) | | *p* value |
| --- | --- | --- | --- | --- | --- | --- |
|  | Median | Q1-Q3 |  | Median | Q1, Q3 |  |
| miR-21 | 0.801 | 0.587-0.869 |  | 0.595 | 0.528-0.778 | 0.157 |
| miR-146a | 2.612 | 2.089-2.831 |  | 2.108 | 1.894-2.555 | 0.086 |
| miR-146b | 1.098 | 0.928-1.359 |  | 1.080 | 0.960-1.180 | 0.621 |

Gd-: patients without gadolinium enhanced lesions; Gd+: patients with gadolinium enhanced lesions; Q1-Q3: First quartile-Third quartile

Figure S1. Receiver Operating Characteristics (ROC) analysis of individual (a-c) and combined (d) CSF miR-21, miR-146a and miR-146b to discriminate inflammatory activity

| (a) miR-21 | (b) miR-146a |
| --- | --- |
|  |  |
| (c) miR-146b | (d) miR-21 + miR-146a + miR-146b |
|  |  |

Figure S2. Correlation analysis of deregulated miRNAs and candidate targets mRNA expression in publicly available dataset.

| 1. miR-21 and IRAK-1 expression | 1. miR-146a and IRAK-1 expression |
| --- | --- |
|  |  |
| 1. miR-146b and IRAK-1 expression | 1. miR-21 and EGFR expression |
|  |  |
| 1. miR-146a and EGFR expression | 1. miR-146b and EGFR expression |
|  |  |

miRNA and mRNA expression profile of GSE28487 and GSE28490, respectively from Gene Expression Omnibus (GEO) DataSets (https://www.ncbi. nlm.nih.gov/gds/) was obtained. Scatter plots of correlations between upregulated miRNAs in CSF (miR-21, miR-146a and miR-146b) and their shared target genes (*IRAK-1* and *EGFR*) were presented. r_s_: Rho of Spearman; *p*: p value

Figure S3. Correlation of normalised Ct between CSF and plasma for (a) miR-21, (b) miR-146a and (c) miR-146b.

| a) miR-21 | b) miR-146a |
| --- | --- |
|  |  |
| c) miR-146b |  |
|  |  |

Scatter plots showing the relationship between CSF and plasma levels for the deregulated miRNAs. r_s_: Rho of Spearman; *p*: p value
